# High-Throughput Corn Image Segmentation and Trait Extraction Using Chlorophyll Fluorescence Images

**DOI:** 10.34133/2021/9792582

**Published:** 2021-07-21

**Authors:** Augusto Souza, Yang Yang

**Affiliations:** Institute for Plant Sciences, Purdue University, West Lafayette, IN, USA

## Abstract

Plant segmentation and trait extraction for individual organs are two of the key challenges in high-throughput phenotyping (HTP) operations. To address this challenge, the Ag Alumni Seed Phenotyping Facility (AAPF) at Purdue University utilizes chlorophyll fluorescence images (CFIs) to enable consistent and efficient automatic segmentation of plants of different species, age, or color. A series of image analysis routines were also developed to facilitate the quantitative measurements of key corn plant traits. A proof-of-concept experiment was conducted to demonstrate the utility of the extracted traits in assessing drought stress reaction of corn plants. The image analysis routines successfully measured several corn morphological characteristics for different sizes such as plant height, area, top-node height and diameter, number of leaves, leaf area, and angle in relation to the stem. Data from the proof-of-concept experiment showed how corn plants behaved when treated with different water regiments or grown in pot of different sizes. High-throughput image segmentation and analysis basing on a plant's fluorescence image was proved to be efficient and reliable. Extracted trait on the segmented stem and leaves of a corn plant demonstrated the importance and utility of this kind of trait data in evaluating the performance of corn plant under stress. Data collected from corn plants grown in pots of different volumes showed the importance of using pot of standard size when conducting and reporting plant phenotyping data in a controlled-environment facility.

## 1. Introduction

Corn is an essential crop because of its high yield and its suitability for a variety of different purposes, such as for food, energy consumption, and as animal feeds [1]. It is the largest cereal crop in the US, with a production of approximately 350 thousand metric tons in 2019 [2]. The demand for this cereal is ever increasing in order to meet the growing needs for a rapidly growing world population which could reach 9 billion in the coming decades [3]. There have been many efforts in identifying, improving, and breeding corn with novel traits to alleviate the impacts caused by changes in climate and environmental conditions [4].

Morphological changes are among many sophisticated adjustments plants made in reaction to drought stress and have been extensively studied [5]. However, due to the lack of high-throughput phenotypic trait extraction, especially at the leaf and stem levels [6, 7], there has been a significant gap between the expertise in the molecular aspects of drought stress response in corn and that in the whole plant physiology, morphology in corn under drought stress, even with the tremendous interests in both the agriculture industry and academia to look for approaches to improve drought-tolerance in corn.

Until recently, the majority of plant phenotype-related data have been collected manually and in destructive ways, which were low throughput, time-consuming, labor intensive, and were not as reliable nor consistent as desired. Data collected these ways were also limited to specific growth stages and were not able to reflect the overall impact of drought stress on corn plants through the complete growing period [8, 9]. There have been extensive interests in developing automated, high-throughput solutions to automatically extract and analyze plant traits at high spatial and temporal resolutions [7, 9, 10].

High-throughput phenotyping (HTP) integrative phenotyping facilities offer researchers the capabilities in continuous, automatic, nondestructive, and multimode measurements of plant traits, such as those related to plant development and growth, and the morphological and physiological traits that are related to plant reactions to abiotic/biotics stresses [10, 11]. Even though not all the technologies established in the HTP facilities are necessarily novel, such as visible (e.g., RGB) or hyperspectral imaging technologies, the high-throughput capacity and noninvasive capability of this kind of facilities provide the opportunity to measure large amount of plants at high spatial and temporal resolutions.

The Ag Alumni Seed Phenotyping Facility (AAPF) at Purdue University is an automated HTP facility that is able to facilitate high-throughput, nondestructive measurements on multiple traits of different crops. It has a growth chamber that is capable of supporting 256 plants simultaneously under the highly precisely controlled environmental conditions. The irrigation management system is able to provide each plant fertigation solutions of different recipes at different amount with high precision. The color (RGB) imaging booth at the AAPF is capable of taking the top and side-view images of plants up to five (5) meters in height. Combining this RGB imaging system with an automated image analysis data pipeline makes it possible to conduct autonomous measurements on various corn traits, such as plant height, width, and projected area.

One of the key steps in applying the RGB images for plant phenotyping is segmentation [12], which is to separate the targets of interest from the background. Various methods ranging from simple image processing to sophisticated artificial intelligence algorithms have been implemented to facilitate image segmentation in different applications [13, 14]. A majority of these methods, such as the color index-based technologies, are based on finding the difference in the color between the target of interests and the background, such as the Excess Green Index (ExG) [15, 16], the Excessive Red Index (ExR) [17], combined indices 1 and 2 [18, 19], as well as the Modified Excess Green Index (MExG) [20], or other color quantification systems, such as the decision tree developed using HSV values [21]. However, the majority of these methods have run into challenges when the color in the target plants or background changes due to differences in the species, varieties, or growth stage [22] or as lighting conditions change, shadows present, or background scene varies [23], complicating the plant segmentation in an HTP facility's automated image processing pipeline.

On the other hand, it has been well established that part of the light energy absorbed by the chlorophyll of a green vegetation plant can be reemitted as chlorophyll fluorescence [24]. The spectrum of the chlorophyll fluorescence is different from that of the absorbed light. Chlorophyll fluorescence imaging (CFI) has been extensively leveraged in assessment of plant reaction and performance under wide varieties of abiotic or biotic stresses [25–27]. CFI has also been leveraged for plant segmentation from the background, but not in HTP systems and only for the plant leaf [28]. However, there are very limited reports on leveraging this kind of signal for automated high-throughput image segmentation in large-scale plant phenotyping studies.

Another key bottleneck in leveraging imaging technologies, such as the RGB imaging systems, in automatic image-based high-throughput plant phenotyping is the extraction of phenotypic traits or the lack of trait extraction so to speak, for specific organs of a plant, such as the stem and leaves of a corn plant. In many typical image analysis tools for plant phenotyping [9, 10], the maximum vertical extend of the plant from the soil surface or the top of a pot to the top-most tip has been extracted as the height of the plant. In many studies related to corn, it is also necessary to take into account how the length from the soil surface to the node of the top most fully extended leaf [29–32] and leaf angles [6, 33] impact a corn plant's key processes such as radiation and water use efficiency. When a corn plant experiences drought stress, early leaf senescence has also been observed for older leaves at the bottom layer of a plant [34].

In most of the HTP phenotyping facilities, plants are grown in pots of certain volume. In forestry/horticulture, people have been paying attention to pot size to either look for the pot of smallest volume that could still deliver a product with adequate quality [35] . But the reports on the impact of pot size on plant performance in phenotyping related studies have been very sparse [36, 37].

In this report, we intend to introduce an RGB image-based high-throughput plant phenotyping system in which we heavily leverage the plant chlorophyll fluorescence signal in segmentation of corn plants. Additionally, image analysis transcripts have been developed to extract a segment of the stem (considered as part between the surface and top node) and leaf traits of a corn plant. Using the data from a proof-of-concept experiment, we aim to demonstrate the utility of the extracted traits, such as the length of the segmented stem, leaf number, and leaf angle of a corn plant, in assessing the performance of corn plants under different irrigation regimes or grown in pot of different sizes.

## 2. Materials and Methods

### 2.1. Corn Plant Treatment and Ground-Truth Measurements

**Figure 1:**
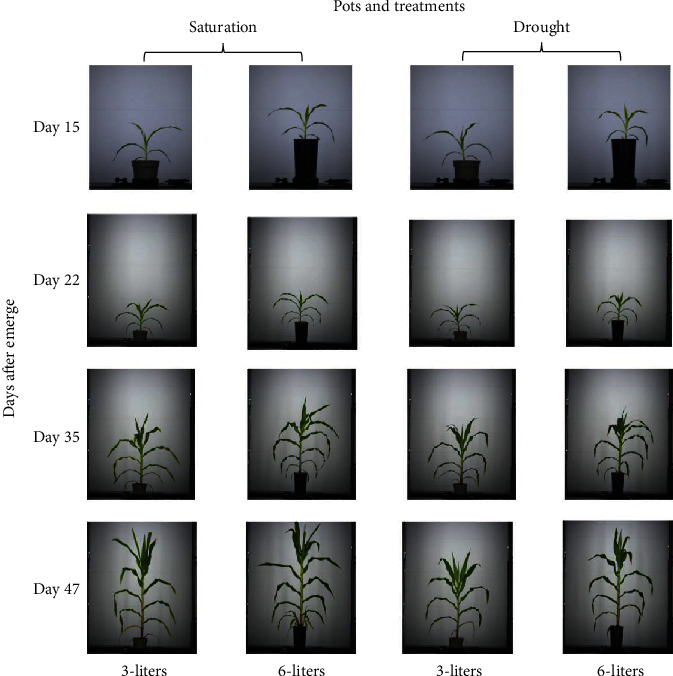
Proof-of-concept experimental design. The experiment had two treatments: saturation and drought. It also used two types of pots with different sizes (image scale 1/12).

The experiment was conducted with 32 corn plants (B73 × Mo17) cultivated in the growth chamber at AAPF until 47 days after emergence (DAE). The potting media used was 50% peat-moss-based potting mix and 50% profile. Plants were grown in pots of two different volumes. The smaller container came with a height equal to 200 mm and a nominal volume of three (3) liters. The taller pots were 400 mm in height at a nominal volume of six (6) liters. A weight-based automated fertigation system managed the irrigation operation. Plants were fertigated daily with two different regimes, with half of the plants being well-watered, and the other half receiving only 50% of the amount of solution provided to the well-watered plants. At each fertigation event, fertilizer solution was added to each pot to reach a predefined target weight. For the six-liter pots, the target weight was set to 6455 grams for the well-watered plants, and 4933 grams for the ones in the drought stressed treatment. For the three-liter pots, the target weights for the well-watered and drought-stressed treatments were 3697 and 2697 grams, respectively. The drought stress treatment was initiated twenty (20) days after emergence. The experimental arrangement is shown in Figure 1.

On DAEs 15, 22, 35, and 47, respectively (in correspond to growth stages at about V4, V6, V8, and V10, respectively), eight plants were harvested, and plant height, the length of the segmented stem, and the stem diameter, as well as the number of leaves, were measured. A carpenter ruler with a half centimeter resolution was used in measuring the plant height, stem segment height, and stem diameter. The plant height was measured from as the vertical distance between soil surface and the top most point in the plant. The stem diameter was measured at several locations and then averaged. The stem height was measured from the soil surface to the top-most collar node. A LI-3100C leaf area meter (LI-COR Biosciences, Lincoln, Nebraska, USA), with 0.1 mm^2^ resolution, was used to measure the leaf area. Initially, the area of each fully developed leaves was measured and annotated individually so it could be compared to the area measured by the algorithm. The other young leaves were measured following to calculate the total leaf area.

### 2.2. Images Acquisition

The corn plants were imaged daily. Every time when a plant was to be imaged, it was carried into the imaging booth on an automated convey belt and then stood on a rotation table. The numbers of plants that were imaged and the actual amount of images used are shown in Table 1 at their respective DAEs. For whole plant analysis, we used all images except for the ones showing plants with torn or broken leaves. For the leaf labeling algorithm, it was used only the images taken for the eight plants harvested at a particular DAE.

**Figure 2:**
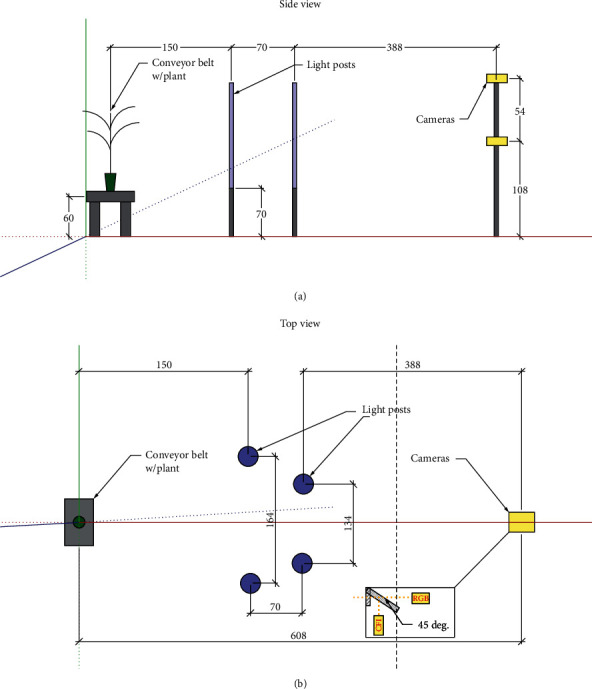
Schematic description within the camera booth: both the (a) side- and (b) top-view descriptions of the camera and light configuration within the imaging booth. Camera box: a mirror set at 45 degrees splits lights into both cameras.

Multiple imaging boxes from ARIS RGB imager (Aris, Eindhoven, The Netherlands) were installed in the imaging booth to acquire side- and top-view plant images, as illustrated in Figure 2. Every imaging box consists of two Basler Ace cameras. The standard RGB camera is a model acA2440-20gc camera with a 5 MP CMOS sensor and provides a 23 fps frame rate. A monochrome camera (acA2440-20gm) with the same sensor resolution and frame rate is equipped with a long-pass filter to capture chlorophyll fluorescence images (zoomed box in Figure 2(b), top view). A blue light emitting diode (LED) array and a white LED array provide the strobing light for image acquisition. Every time when a plant comes into the imaging position on the rotation table, the blue LED array will fire up to excite chlorophyll fluorescence signal from the plant. The monochrome camera is synchronized with the blue LED light strobe for chlorophyll fluorescence image acquisition. The white LED then fires up, with the color camera synchronized to acquire RGB image of the plant. An ARIS provided image registration function makes sure that the images acquired by the two cameras are aligned. To better visualize the camera system, a virtual tour is provided showing the AAPF capabilities (https://ag.purdue.edu/cepf/).

For data reported in this manuscript, two side-view imaging boxes were used, namely, the side-small camera box, which is dedicated to acquiring images for plants shorter than 20 centimeters, and the side-bottom camera box for plants taller than 20 centimeters. A third camera box was used to take top-view plant images. The top-view images were leveraged to extract information on the orientation of a plant, so that when the side-view images were taken, the plant would always be rotated in a way in which it would face the side-view cameras with the broadest observable side. This angle was calculated according to the longest axis within the top-view image plant convex hull and the horizontal *x*-axis. The symmetry of the corn plant architecture was leveraged to increase the number of images and sample size. Images of the corn plant orientation rotated at 180° of the initial angle were also used for both whole plant and leaf labeling analysis. For most of the examples, it was similar to a mirrored image of the original one selected.

### 2.3. Image Analysis

**Figure 3:**
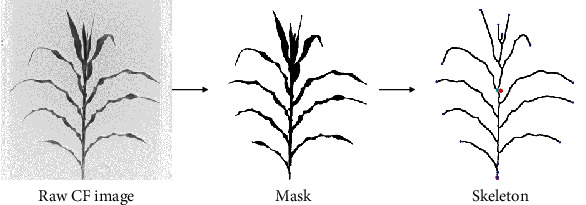
Segmentation and skeletonization: the image analysis processes started with building the binary mask using the gray-scale chlorophyll fluorescence (CF) images; subsequently, the skeleton was created as a two-dimensional wireframe of the plant (image scale 1/4).

**Figure 4:**
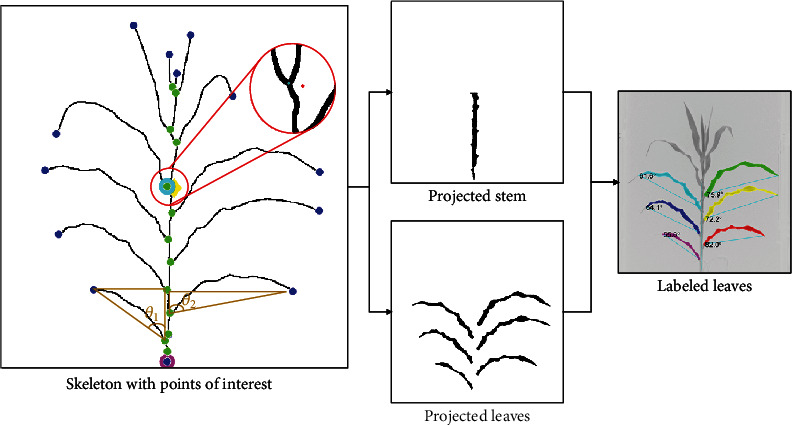
Canopy traits extraction: extraction based on the points of interest calculated in the skeleton. First, the segmented stem and leaves were labeled; features (traits) of the labeled organs were then calculated (image scale 1/4). Skeleton with points of interests: the blue markers are the endpoints and leaf tips. The purple one is the stem start, and the cyan one is the top node, considered the end of the segmented stem. The green points are the nodes of the plant or branch points. The yellow diamond-shape marker is the center of mass of the skeleton. The angle between leaf tip and stem was calculated based on the two extremity points of the leaf, as exemplified by *θ*_1_ and *θ*_2_ (image scale 1/2).

The image analysis processes started with creating the segmentation mask using the monochrome chlorophyll fluorescence image. The mask was then skeletonized to facilitate identifying the points of interests, such as leaf tips, branch points, the coordinates of the stem extreme points, and the center of mass of the skeleton (Figure 3). These points of interest assisted in establishing the key segment of the stem and leaf traits (Figure 4). For this study, the corn plant stem was defined as the point from the surface to the node with the youngest fully developed leaf, based on the Leaf Collar method to determine the vegetative stage of a corn plant [38]. This is a traditional method to define the stem height in corn breeding, and this manuscript introduces how to estimate the segment of this trait using computer vision techniques.”

Skeleton pixels that belonged from the soil surface to the top node were identified in order to establish the mask for the segmented stem and to calculate its dimensions, i.e., stem height or top node height and stem diameter. The mask of the stem was in turn subtracted from the overall plant mask of leaving only the leaves. The leaves were then labeled accordingly, and the traits of each leaf were calculated. All image analysis processes were implemented using MATLAB version 2019a (Mathworks, Natick, Massachusetts, USA) in a laptop computer with 64-bit Windows 10 Image v9 operating system (Microsoft Corporation, Redmon, Washington, USA) with an Intel Core i5-8250U CPU @ 1.60 GHz, with 8.00 GB of RAM. The following sections explain in details the steps taken to calculate every feature included in this manuscript.

### 2.4. Segmentation and Skeletonization

Using the collected chlorophyll fluorescence image, the Otsu's algorithm [39] was adopted to establish a threshold to separate foreground and background based on the histogram of the gray values in the image. A binary mask was generated using this threshold: any pixels with values smaller than the threshold were considered background and assigned a value zero (0); otherwise, the pixel was classified as the plant and assigned a value one (1). A sequence of binary image morphological operations, including opening and closing operations, followed the binarization operation to minimize undesirable noises before establishing the final mask of the plant. The single-pixel wireframe structure or skeleton of the plant was generated by skeletonization of the mask. The skeletonization was followed by a trimming operation to remove small pixel branches to ensure a smooth and continuous skeleton (Figure 3).

### 2.5. Traits of the Whole Plant

Using the established mask, traits such as the side-projected plant area, the overall plant height, and width of the plant were calculated. The side-projected plant area was calculated as the sum of the pixels in the mask. The plant height was calculated as the vertical length of the rectangle which enclosed the plant mask. Initially, these measurements were represented in number of pixels. A set of preestablished calibration function developed by the camera vendors then converts the pixel numbers into physical units (e.g., cm and cm^2^).

#### 2.5.1. Endpoints and Branch Points

The endpoint and branch points of the plant skeleton were established to characterize the plant and its organs (Figure 4). The endpoints of the skeleton, except for the one at the very bottom of the image, which was considered as the stem start point if it was also located at the middle plane which separated the mask into two equal halves, were labeled as leaf tips. If a leaf tip droops lower than the top of the pot, its coordinates consequently would be lower than those of the stem start point. However, since the coordinates of this leaf tip do not lie on the center line which separated the plane into two more or less equal halves, it would not be counted as the start point of the stem, but rather a leaf tip. The established end and branch points were further leveraged to identify the pixels of a stem. In Figure 4, the leaf tips are highlighted by round blue markers, while the stem start point is represented by a large circular magenta marker.

#### 2.5.2. Stem Dimensions

The top node height was calculated as the distance between the start- and endpoint of the segmented stem. The endpoint of the stem was identified leveraging the established branch points. To do so, the center of mass of the skeleton was first established. The branch point that was closest to the skeleton's center of mass was then designated as the top node and endpoint of the stem (Figure 4, zoomed circle), where the plant skeleton center of mass is represented as a red diamond, and the stem end is the blue round marker. Their proximity was used to assume the top node location.

**Figure 5:**
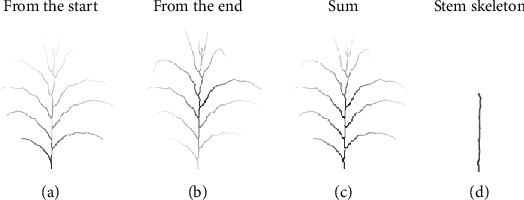
Procedure to identify the segmented stem path. The gray-scale wireframes at (a) and (b) represent, respectively, the distance between the stem start and top node: the darker is the skeleton path, and the smaller is the distance, in pixels, between these extremity points and the other skeleton points. The sum of these two outputs is illustrated in (c), and its regional minimum is shown in (d), which can be interpreted as the stem skeleton and final path (image scales 2/5).

To establish the mask of the stem, the geodesic distances between all the other pixels of the skeleton and the start (Figure 5(a)) or top node (Figure 5(b)) were calculated. The two sets of geodesic distance were summed up as shown in Figure 5(c), of which the gray-scale intensity represents the distance between a pixel and the two extreme points of the stem, i.e., the brighter the pixel, the further away it is from the start- and endpoint of the segmented stem. Thereafter, a search was conducted to identify the set of connected pixels that would construct the path with the minimal distance between the start- and endpoint of the stem. The output was considered as the path of the stem in the skeleton (Figure 5(d)).

The distance transform of the plant mask was subsequently conducted following the method reported by Brichet et al. [40]. Then, tracking the stem path, the distances between each pixel on the stem path and the pixel to its right (or left) on the edge of the stem mask were calculated and designated as the radius of the stem along the stem path. To minimize noise, this radius array was then filtered by a median filer. The outcome was the representative radius of the stem. The diameter of the stem was then established by multiplying the radius by two.

After both the stem path and the stem radius were established, all the pixels belonging to the stem were identified by searching along both side of the stem path for adjacent pixels with distance smaller than or in equivalent to the stem radius. After all the pixels belonging to the stem were labeled, the segmented stem was subtracted from the plant mask, leaving only the leaves. To this point, two distinct masks had been created for a corn plant: one of the segments of the stem and another for the leaves (Figure 4).

### 2.6. Leaf Traits

The number of fully expanded leaves was counted in the mask, where they were first labeled. To correctly count them, the cluster of the leaves in the whorl (at the top part in a leaf mask) was removed, since they are not considered as fully developed/expanded, by eliminating the labeled blob with the highest center of mass. The rest of the leaves in the mask were labeled again afterward: first, we calculated the center of mass for each remaining labeled leaf. The one with the lowest center of mass was labeled as number one; the leaf with the second lowest center of mass was labeled as number two and so on until all fully expanded leaves were labeled. In this way, it was making sure that the youngest expanded leaf was always labeled as the top most expanded leaf. After each fully expanded leaf was labeled, the side-projected area of each leaf was calculated as the individual sum of pixels and then converted into cm^2^ using a calibration function previously established. The angle between the leaf tip and the stem was calculated in the way as illustrated in Figure 4.

## 3. Results

### 3.1. Image Analysis Results

Using the previously described computer, the algorithm took 786 seconds to process 143 images and write the results in a spreadsheet file when analyzing the holistic traits, while for the leaf labeling, for 38 images, it took 216 seconds. Some plants had two images for the same DAE since they had picture with two orientations: 0° and 180°. Some images were removed due to damaged leaves or stems and tillers and overlapped leaves that hampered the algorithm.

**Figure 6:**
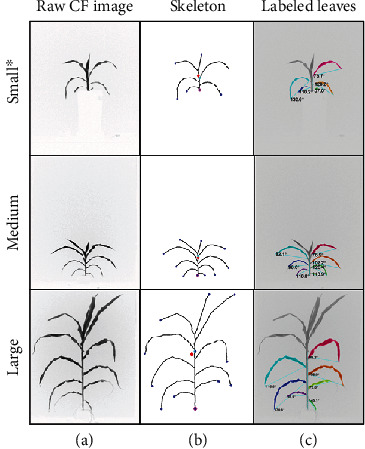
Corn phenotyping for plants in different sizes: raw images for plants with diverse sizes along with their skeleton and labeled leaves to check the algorithm efficacy. ^∗^For the small plant, the side-small camera was used, while the side-bottom was used for the other two (image scale 1/6).

Using the chlorophyll fluorescence signal, we were able to reliably segment images of very young or well-developed corn plants (Figure 6). Neither the variations in plant size or age of a plant, nor the differences in leaf color, caused any deterioration in the script's capability in isolating the pixels that belonged to the plants from the background pixels. The reliable segmentation facilitated the success in skeletonization of the mask and in defining the start- and endpoint of a stem (Figure 6(b)) on plants of different sizes. With the extreme points of a stem established, the script was then able to isolate the pixels belong to a stem and label leaves thereafter, for plants of different sizes and different number of leaves (Figure 6(c)). The traits of the leaves, such as the leaf area and angle between leaf tip and the stem, were then successfully calculated for all samples during the experiment.

**Figure 7:**
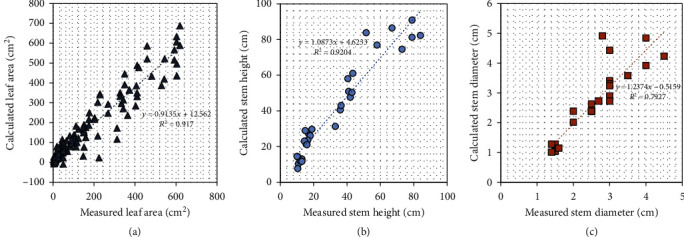
Image-based vs. ground-truth measurements. The total area of the fully (a) expanded leaves, (b) the segmented stem heights, and (c) the stem diameter calculated (*x*-axis) using the acquired images was comparable to the ones collected through manual ground-truth measurements (*y*-axis).

Comparison between ground-truth data and image-extracted measurements showed good correlation in measurements on area of fully expanded leaves (Figure 7(a)) and the top node height (Figure 7(b)), with *R*^2^ values ranging from 0.92 to 0.97. The area of fully expanded leaves ranges from 10 cm^2^ for plants at V3/4 to close to 600 cm^2^ for plants at V10. The image-extracted area slightly underestimated the actual measurements when the plants became older, but in general, the image-based measurements followed the ground truth very well. The segmented stem heights were approximately 10 cm for younger plants at DAE 15 and were around 80 cm at DAE 47 (V10). The image-estimated stem heights also agreed with the ground truth better when plants were at relatively earlier stages.

### 3.2. Proof-of-Concept Experiment Results

**Figure 8:**
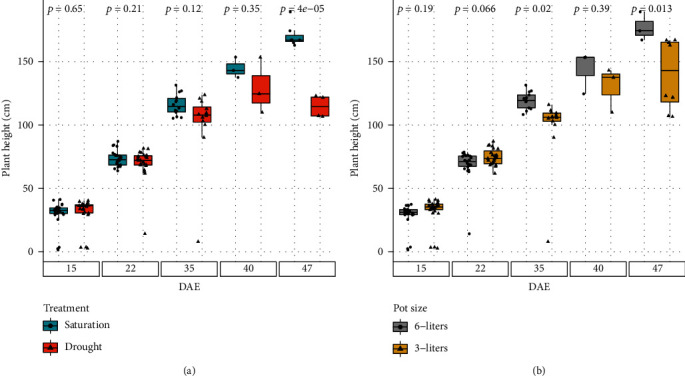
Plant height: the plant height, in centimeters, for the four different scenarios of the proof-of-concept experiment for corn plant at different ages. Above the box plot is the *p* value for a two-sample *t*-test, where the two samples with *p* < 0.05 are statistically different from each other.

Differences in irrigation treatment affected the total height of the corn plants during the experiment. As show in Figure 8(a), early in the experiment, there were no significant differences in plant height between well-watered and drought-stressed plants. As the experiment progressed, the differences in plant height became progressively more evident, even though not statistically different at some stages. After DAE 35, the plants in the well-watered group kept growing taller, but the median height in the drought-stressed group did not increase much, and the plant height has a lot of variance for this day. By DAE 47, the difference in total plant height for plants in different irrigation treatments was very significant. The median height of the well-watered plants was around 168 cm while that of those in the drought-stressed group was at 115 cm.

The volume of the pot also showed significant effects on the total height of the corn plants (Figure 8(b)). The separation between plant heights in different pot volumes started to be observable on DAE 35. As the plants grew larger, the difference became more significant. By DAE 47, the plants grown in the six-liter pots were close to 30 cm taller than the ones grown in the three-liter pots.

**Figure 9:**
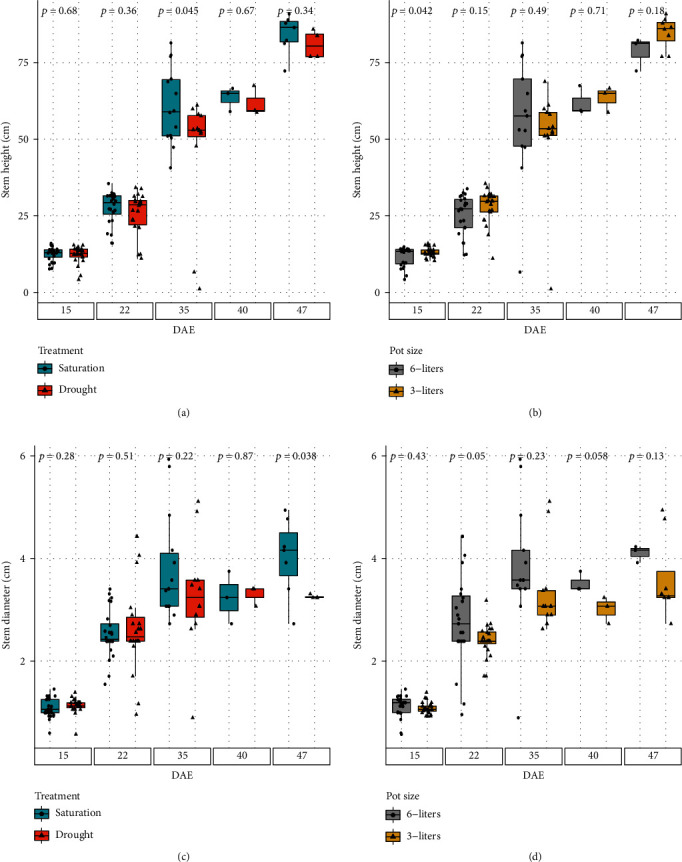
Segmented stem dimensions: (a, b) height and (c, d) diameter of the stem segment for saturated and drought treatments and different pot sizes at different DAEs. Above the box plot is the *p* value for a two-sample *t*-test, where the two samples with *p* < 0.05 are statistically different from each other.

However, the differences in irrigation treatment or pot volume did not result in differences in height of the segmented stems, besides for a couple days. This stem heights of the plants in both different irrigation treatments or pot volumes were very similar, starting at about 10 cm on DAE 15 and ended with 80 cm on DAE 47. No statistically significant differences were observed during the entire experiment (Figures 9(a) and 9(b)), besides for DAE 35 for the irrigation treatments and DAE 15 for the pot difference. No statistically significant differences were observed in the diameter of the plant stalk in different irrigation treatment (Figure 9(c)) or pot volume (Figure 9(d)), with the exception of DAE 47 for the different irrigation regiments and DAEs 22 and 40 for the different volume pots. The thinnest diameter started at about 1.2 cm in average at DAE 15 and ended at around 4 cm on DAE 47. Even though there were minor differences among the mean diameter values of different groups, the large variations in the measurements rendered the differences almost insignificant.

**Figure 10:**
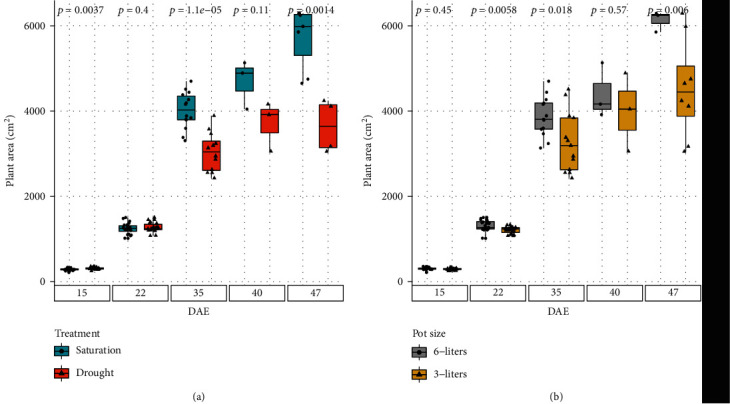
Plant area: the plant area, in cm^2^, for all four treatments that corn plants were cultivated during 47 days. Above the box plot is the *p* value for a two-sample *t*-test, where the two samples with *p* < 0.05 are statistically different from each other.

The differences in irrigation treatment and pot volume affected side-projected plant area. The median side-projected area of the drought-stressed plants started to lag behind those of the well-watered plants on DAE 35 (Figure 10(a)). By DAE 47, the side-projected plant area of the well-watered plant reached 6000 cm^2^ while those in the drought-stressed group were only slightly above 3500 cm^2^. The averaged side-projected area of the plants in the larger pots started to separate from those of the ones in the smaller pots on DAE 35 too (Figure 10(b)). By DAE 47, the average plant size in the six-liter pot was 6500 cm^2^, which was 2000 cm^2^ larger than the ones in the three-liter pot.

**Figure 11:**
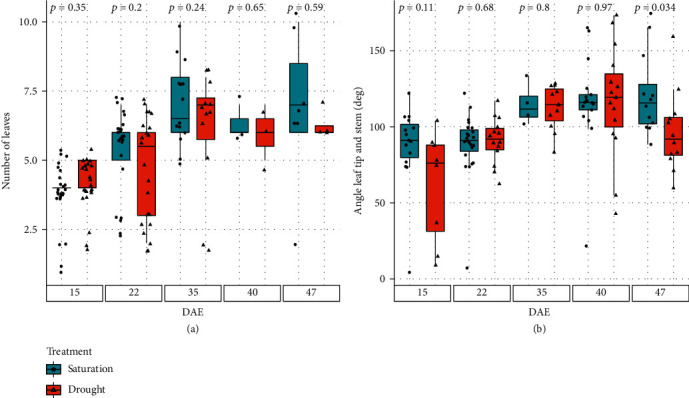
Number of leaves and angle between stem and leaf tip: experimental results for (a) the number of leaves and (b) the leaf angle to the stem for the water regiment treatments. Above the box plot is the *p* value for a two-sample *t*-test, where the two samples with *p* < 0.05 are statistically different from each other.

The number of expanded leaves, the area of the fully expanded leaves, and the angle between tips of fully expanded leaves and the stem are shown in Figure 11. Early on in the experiment, no differences in number of fully expanded leaves were observed (Figure 11(a)). Yet by DAE 47, the well-watered corn plants in median had two more fully expanded leaves than the drought-stressed ones. The average angle between the leaf tip of the fully expanded leaves was similar between different irrigation regimes early on during the experiment. But, by DAE 47, the average leaf tip angle of the well-watered plants was 115 degrees, while that of the drought stressed ones were around 85 degrees (Figure 11(b)).

## 4. Discussion

The configuration of multiple imaging boxes in the RGB imaging booth provides versatility to accommodate the needs of researches which bring wide variations on the size/age of plants, from small seedlings of corn, sorghum, tomato, or tobacco, or even small model plants, such as *Arabidopsis*, to large ones, such as the fully grown hybrid corn or sorghum plants. The RGB imaging systems in the AAPF take full advantage of the fact that the fluorescence signal uniquely belongs to a green vegetation with chlorophyll [24]—no matter the amount of chlorophyll, thereby no matter the greenness of the target—by synchronizing the blue strobing light (for the excitation of the chlorophyll fluorescence signal) and the monochrome camera equipped with a long-pass-filter. In the gray-scale fluorescence images acquired by this imaging system, the pixels that belong to a plant (in this case a corn plant) are much brighter than the pixels in the background, which emit hardly any light in the chlorophyll fluorescence waveband. Segmenting this kind of image with the Otus' algorithm, no predefined threshold value is needed, no matter the color (or hue) of the target plant. This system is therefore much more robust than the color- or color index-based methods in handling changes in target color [22] or variation in lighting conditions [21], making it a preferable option in segmentation of images for high-throughput automatic image-based phenotyping for plants of different species, varieties, or developmental stages. However, there are limitations using CFI and the Otsu's method if some parts of the plant have considerably lower chlorophyll levels. Even though this was not the problem for this study, it could be an issue for aging corn plants, especially in the reproductive stages and physiological maturity. One possible future improvement could be to apply the automatic segmentation for different sections or windows in the image and avoid applying the same threshold for the entire plant.

The shape of the corn stem, especially the one of well-developed plants, has been reported to be a tapered elliptical cylinder [40], which means the cross-section profile of the stem is an ellipse, and the cross-sectional area gradually reduces from bottom to the top of the stalk. In manual measurements, we tried our best to identify the minor axis of the elliptical cross section of the stem. However, in the imaging procedure, we were only able to make sure the widest side of the whole plant, always facing the side-view cameras. But we had no control over which side of the stem was facing the camera when an image was taken. When the major axis of the elliptical cross section was facing the camera when imaging, the calculated stem diameter would overestimate the measurements. This could explain some of the overestimation we have observed in the stem diameter calculation for older corn plants.

The key step in our efforts to extract the traits for corn plants, such as the number of fully expanded leaves and the top node height, was the skeletonization of the plant mask. We established a simple yet effective way in identifying the endpoint by choosing the top most branch point that is closest to the center of mass of the skeleton. The implementation of this algorithm was elegant and relatively straight forward, and the method did not come with high computational demand. A potential pitfall we noticed when leveraging this method was that when leaves overlapped extensively, the risk of establishing false branch points in the skeleton also increased significantly, thereby misleading the algorithm in establishing the tips of the leaves or in locating the start- or endpoint of the segmented stem. Because of this reason, it was challenging to leverage this method to process images of older corn plants or corn plants with large amount of tillers. The images processed for this report were of the plants grew up to 47 DAE. For plant of ages beyond 47 DAE, we noticed that the number of false branch points surged. Also, when a plant is producing ears, then the position of the center of mass in the skeleton of the plant changes, and the methods we report here could not work as reliably in that situation either. So, for corn plant at more advanced phenological stages, yet more robust methods for high-throughput image analysis are desirable. To that end, 3D imaging acquired using LiDAR [7, 9], and the recent developments in applying deep learning in machine vision feature extraction, such as what was reported in [41], seem to be promising. Even for the deep learning methods though, what we have developed here can be used in high-throughput labeling of the training data set for younger plants.

The number of fully expanded green leaves has been an important trait in corn. Yet, it has been challenging in labeling and counting individual leaves in corn plant phenotyping, even though the architecture of a corn plant is relatively simpler comparing to other crops. The performance of our leaf counting efforts was acceptable for plants at V4 and V6. However, as the plants grew older, the performance declined, as seen in Table 2.

The lower number of leaf tips in the drought-stressed plants reflected the earlier senescence of lower leaves in drought-stressed corn plants [29, 34]. One limitation of our algorithm was to deal with occluded, overlapping, and drooping leaves, especially for corn plants at V8 and later. For all images, the algorithm was able to calculate the canopy morphological characteristics, including segmented stem height and diameter. However, as observed in Table 2, there was a discrepancy in the number of leaves and the correct labeling of these plants at a later stage. Thus, this algorithm may not be the best fit at this vegetative stage or later. The automatic plant orientation implemented is similar to another high-throughput phenotyping facility [42]. However, this methodology could lead to errors when measuring traits in abnormal and nonsymmetrical corn plants by occluding leaves or other organs and minimizing plant area. A possible solution is to use postprocessing techniques using images from several angles to identify the optimal one that shows all the aboveground parts of the plant instead of relying only on the top-view image. Another area that can lead to erroneous interpretation is stem identification. Our method of identifying the top node worked for plants at the studied vegetative stage; however, this could change with the morphology of the corn plant at later stages. Thus, it needs more investigation to identify the top node using the plant center of mass and estimate the segmented stem for older corn plants. Similarly, the order of the leaves was also determined by their center of mass that could result in the wrong order if younger leaves are drooping and their center of mass is below the older leaves center, for example. A future possibility for improvement is to use the Cartesian coordinates of the branch points after the leaves were labeled to determine their correct order.

The extracted traits of corn plants, especially the traits of individual organs (stem and leaves in this case), proved to be valuable tools to ascertain corn plants' response to drought. The observed reduction in whole plant height in our data seemed to agree with the documented reduction in leaf expansion under drought [30, 32]. The statistically similar stem heights in different watering regimes corroborated the rationale that the reduction in expansion of leaves, especially the unexpanded ones in the whorl, was the main reason for the reduction in plant height. The lower number of leaf tips in the drought-stressed plants reflected the earlier senescence of lower leaves in drought-stressed corn plants [29, 34]. Rolling in leaves, especially the top ones, has also been identified as a useful drought avoidance mechanism in corn [33, 43]. Rolling leaves reduce the effective leaf area, which can also contribute to the reduction in side-projected area of the corn plants. Rolling in a leaf also would make a leaf more upright, thereby decreasing the angle between the stem and the leaf [43]. The relatively smaller leaf angle of the drought stressed plants in comparison to that of the controls observed in our experiment could have been caused by the leaf rolling as drought stress developed.

It is worth to point out that the established capability in extracting this segment of the stem height, as demonstrated here, provides us a very valuable tool for in-depth ascertaining of the component in a plant's reaction to drought stress. In the case of B73 × Mo17, our data shows that the leaf elongation was more sensitive to mild- to medium-drought stress than the elongation of the stem. The observation that there were no reduction in stem heights in the drought-stressed plants contradicted early reports in which drought stress starting at early vegetative stage significantly reduced plant height or stem height [29, 34, 44]. However, it has been pointed out in another study that different varieties react to drought differently [44]. The stem elongation of the corn variety tested in our experiment could be more tolerant to relatively milder stress. Furthermore, the drought stress induced in our experiment may not have not as severe as what has been reported; therefore, the established drought stress was not sufficiently severe during the experiment to affect the stem elongation, thereby the top node height, of the drought stressed plants.

The impacts of pot volume in the size of the plants show the importance in considering the pot volume in experiment design and in documenting the size of pot when reporting the results of phenotyping studies. It has been reported that the volume of a pot significantly affects the amount of water available for crop consumption [36]. It is also believed that root confinement may cause growth retardation [31], with reduced leaf expansion rate and reduced photosynthesis as the consequences [45, 46]. Therefore, when comparing plant phenotyping data from different facilities or from different rounds of experiments from the same facility, the volume of pot used in the experiment should be a key consideration. It is very desirable to establish a set of standard pot size for HTP facilities in controlled-environment facilities.

There are similarities between the guidelines here presented and other HTP systems for corn phenotyping. Still, some key differences were necessary to be implemented for our algorithm to work in our system. The first was the segmentation using CFI that, as previously mentioned, showed us a better segmentation mechanism compared to color-based ones or light-dependent. Zhang et al. used the Excess Green vegetation index that is highly dependent on the plant's color [16]. Also, Choudhury et al. and Khan et al. used background removal, which depends on the light intensity and regular calibration of the system [42, 47]. A convolutional neural network, a more advanced techniques, was leveraged for segmentation [48]; however, it was more practical for the AAPF to adopt the CFI segmentation. Regarding stem coordinate estimation, our approach was different and led to diverse results with a different interpretation compared to these researchers' work when using our data set since we estimated the stem path from surface to the stalk node with the youngest fully developed leaf (top node). They all estimated the end of the stem as the last intersection point of the plant skeleton, although Zhang et al. used the Hough Transform to identify the stem [16]. Using this procedure led to errors in our analysis since it mistakenly estimated overlapping, not fully developed leaves at the top of the plant as intersections and, consequently, the end of the stem. An example of a possibly erroneous classification is shown in Figure 4, where the last branch point (magenta markers) is not as near the stem end. Still, on a related topic, only stem length was estimated by their algorithm, while ours calculated the average diameter as well based on the distance transform calculation. Lastly, we were able to count the number of leaves based on the skeleton and the mask as well, while the other three do labeling based on the skeleton joint and leaf tip points. Nevertheless, it is essential to have diverse methodologies since some can be more suitable than others.

In conclusion, the HTP color-imaging data pipeline at Purdue University's AAPF is introduced in this report. The imaging system takes advantage of the unique chlorophyll fluorescence signal from green vegetation and establishes a high-throughput image segmentation and analysis data pipeline. Methods to extract traits of the stem and leaves of corn plants are also reported. Data collected from a proof-of-concept corn vs. drought stress experiment showed that the segmentation using chlorophyll fluorescence signal was consistent and efficient. The extracted traits of corn stem and leaves also demonstrated their utilities when ascertaining corn plants' reaction to drought stress. Corn growth and trait data when using pots of different volumes emphasized the importance in documenting the pot size when reporting plant phenotyping data from controlled-environment facilities. Ideally, to facilitate comparison of research from different facilities, we suggest to adopt a set of pots of standard volumes. In the future, deeper image analysis could be made using the RGB images for 3D recreation and having volume-related phenotype [49].

## Figures and Tables

**Table 1:** Number of plants and images used for the proof-of-concept experiment and algorithm development for all four days after emergence (DAE).

DAE	Vegetative stage	# plants	# images for whole plant	# images for leaf labeling
15	V4	32	51	12
22	V6	24	48	16
35	V7/V8	16	30	4
47	V9/V10	8	14	5
Total			143	37

**Table 2:** The average number of leaves labeled by the algorithm and the visible manually counted, as well as the Root Mean Squared Error (RMSE), at each vegetative stage.

Vegetative stage	Calculated	Visible measured	RMSE
V4	4.9	4.2	1.4
V6	6.0	6.5	1.9
V8	7.6	8.0	8.1
V10	6.6	7.6	7.7

## Data Availability

The dataset and example images could be given upon request from the corresponding authors.

## Supplementary Materials

Supplementary Materials The supplementary spreadsheet file provides the ground-truth measurements and calculations, such 472 as error and comparisons between the outputs of this work and measurements made in loco.
